# Supplementary material for: Materials on Plant Leaf Surfaces Are Deliquescent in a Variety of Environments
Source: Front Plant Sci. 2022 Jul 12;13:722710. doi: 10.3389/fpls.2022.722710 (PMC9315345; doi:10.3389/fpls.2022.722710)
Supplement: Supplementary file 1 [file Data_Sheet_1.PDF]

Supplementary Material for

Materials on Plant Leaf Surfaces are Deliquescent in a Variety of Environments

E. C. Tredenick [1,2,*], H. Stuart-Williams [1] and T. G. Enge [2]

[1]ARC Centre of Excellence in Translational Photosynthesis, Division of Plant Sciences, Research School of Biology, The Australian National University, Canberra, ACT, 2601, Australia,

[2]Department of Engineering Science, University of Oxford, Oxford, OX1 3PJ, United Kingdom,

[3]Research School of Earth Sciences, The Australian National University, Canberra, ACT, 2601, Australia.

*e-mail: eloise.tredenick@anu.edu.au

Table S1: Salt and oil control mixtures. Samples were dissolved/suspended in de-ionised water and oven dried. The percentage mix is found by weighing the salts before creating the solution. For all the oils, approximately 100 mg was the wet weight before oven drying, most of the oil evaporated in the oven. The mangrove nutrient is mainly NaCl, along with 1295 ppm Mg, 430ppm Ca and 390 ppm K.

Compound	Total dry weight - mg	Percentage mix - %
Mangrove nutrient	21.6	-
NaCl + $CaCl_2$	60.0	41 NaCl, 59 $CaCl_2$
NaCl + $CaCl_2$ + KCl	172.5	12 NaCl, 71 $CaCl_2$, 17 KCl
$CaCl_2$ + KCl + NaCl + $Ca(NO_3)_2$	96.5	37 $CaCl_2$, 11 KCl, 17 NaCl, 35 $Ca(NO_3)_2$
$Ca(NO_3)_2$ +KCl	105.3	11 $Ca(NO_3)_2$, 89 KCl
NaCl + euc oil	49.0	96 NaCl, 4 euc oil
NaCl large	24.4	-
NaCl small	1.1	-
$CaCl_2$ large	35.8	-
$CaCl_2$ small	2.1	-
Euc oil	2.7	-
Tea tree oil	0.9	-

S1 Chloride and Sulphate Presence Test

To test for the presence of chloride (Cl^-) and sulphate (SO_4^{2-}), the most abundant anions in sea-water[1], 2 mL of liquid was removed from the 60 mL leaf sample washes destined for the sorption experiment. 1 mL aliquot of solution placed in clean 2 mL glass vials was used to identify chloride and sulphate non-quantitatively. For chloride, a few drops of dilute nitric acid followed by a few drops of silver nitrate ($AgNO_3$) in solution (2% (w/v)); were added to the 1 mL sample. After 10 minute the presence of a precipitate was recorded. The silver nitrate test had the unexpected effect of turning some samples yellow/brown, indicating the presence of oils such as *Eucalyptus* oil and silver nanoparticles may have caused this coloration [2]. Ag^+ is known to react with lipopolysaccharides and amino acids. For the sulphate test, the same procedure was conducted but a few drops of barium chloride solution (2% (w/v)) followed after 10 minutes by a few drops of dilute hydrochloric acid.

S2 GAB isotherm

We consider the Guggenheim, Anderson, and de Böer (GAB) isotherm[3, 4, 5, 6] for fitting a selection of samples. The GAB isotherm describes water adsorption as a monolayer that can form multilayers at high humidities. It is formulated by applying the Langmuir isotherm to each layer (evaporation and condensation can occur only from or on exposed surfaces) and is at equilibrium where the rate of adsorption equals the rate of desorption. The GAB isotherm is as follows:

$$\Delta w = \frac{w_S\ \beta\ k\ RH}{(1-k\ RH)\,(1+k\ (\beta-1)\ RH)}, \tag{S1}$$

where Δw is the percentage weight increase above dry weight at each relative humidity as a percentage, RH ($RH = a_w \times 100\% = p/p_0 \times 100\%$), w_S is the monolayer of water adsorbed per solid, β is the equilibrium parameter of adsorbed water or the interaction energy between water and solid and k represents the difference in free enthalpy of the water molecules in the monolayer and layers above the monolayer, where β and k depend on temperature with the Arrhenius equation and k is always less than 1. The fitting and analysis were performed using MATLAB® (Mathworks, U.S.A.).

References

[1] Bryan, C. R. & Schindelholz, E. J. Properties of brines formed by deliquescence of sea-salt aerosols. In *CORROSION 2018* (OnePetro, 2018).

Table S2: GAB parameters for Figure 4, fitted with Equation (S1)

Sample	w_S	β	$k \times 10^{-3}$	R^2 - %
Cabinet mangrove	46.5	1.5	9.71	98.6
Brackish mangrove	43.4	0.64	9.72	99.0
Mangrove nutrient	162.2	0.098	8.88	99.8
NaCl large	424.4	0.0081	9.36	99.3

Table S3: Operating parameters for ICP-OES measurements.

Parameter	Value
Read time	20 s
Replicates	3
Sample uptake delay	25 s
Rinse time	30 × 2 s (0.5 M HNO_3 & MQ)
Stabilization time	10 s
Pump speed	12 rpm
Fast pump	ON
RF power	1.5 kW
Aux flow	1.0 L min^{-1}
Plasma flow	12.0 L min^{-1}
Nebulizer flow	0.7 L min^{-1}
Viewing mode	SVDV
Viewing height	5 mm
Background correction	FACT
Number of pixels	2
Quality control	1 $\mu g\ g^{-1}$ multi element
Analytes	Al (396.152 nm); B (249.772 nm); Ca (422.673 nm); Cu (327.395 nm); Fe (238.204 nm); K (766.491 nm); Mg (279.553 nm); Mn (257.610 nm); Na (589.592 nm); P (213.618 nm); Si (251.611 nm); Sr (407.771 nm); Zn (213.857 nm)
Drift correction	C (193.027 nm); Ar (565.070 nm, 645.918 nm, 675.283 nm, 699.217 nm, 704.096 nm)

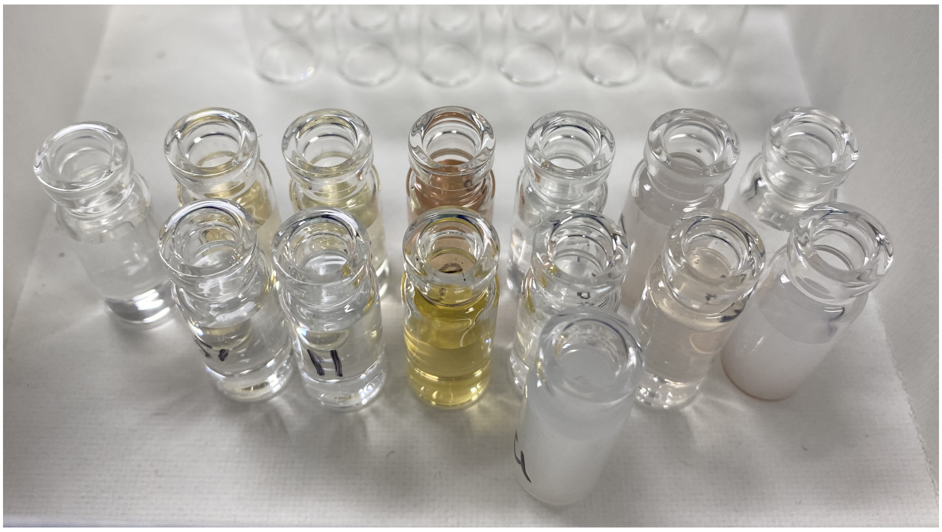

Figure S1: Silver nitrate test for the presence of Cl^-. Samples that reacted formed a milky white precipitate of AgCl. These were Brackish mangrove, Brackish Eucalyptus and Cabinet mangrove. This test also showed the presence of nanoparticles related to Eucalyptus oils with a change in colour from clear of milky to yellow to brown. All 5 Eucalyptus samples changed colour. The darkest colour (copper brown) was Town euc no rain, the second was Town euc rain (dark yellow). The vial in the foreground is the $CaCl_2$ control.

[2] Pourmortazavi, S. M., Taghdiri, M., Makari, V. & Rahimi-Nasrabadi, M. Procedure optimization for green synthesis of silver nanoparticles by aqueous extract of Eucalyptus oleosa. *Spectrochimica Acta Part A: Molecular and Biomolecular Spectroscopy* **136**, 1249–1254 (2015).

[3] Guggenheim, E. A. Applications of statistical mechanics (1966).

[4] de Boer, J. The dynamic character of chemisorptions (1953).

[5] Anderson, R. B. Modifications of the Brunauer, Emmett and Teller equation. *Journal of the American Chemical Society* **68**, 686–691 (1946).

[6] Timmermann, E. O. Multilayer sorption parameters: BET or GAB values? *Colloids and Surfaces A: Physicochemical and Engineering Aspects* **220**, 235–260 (2003).

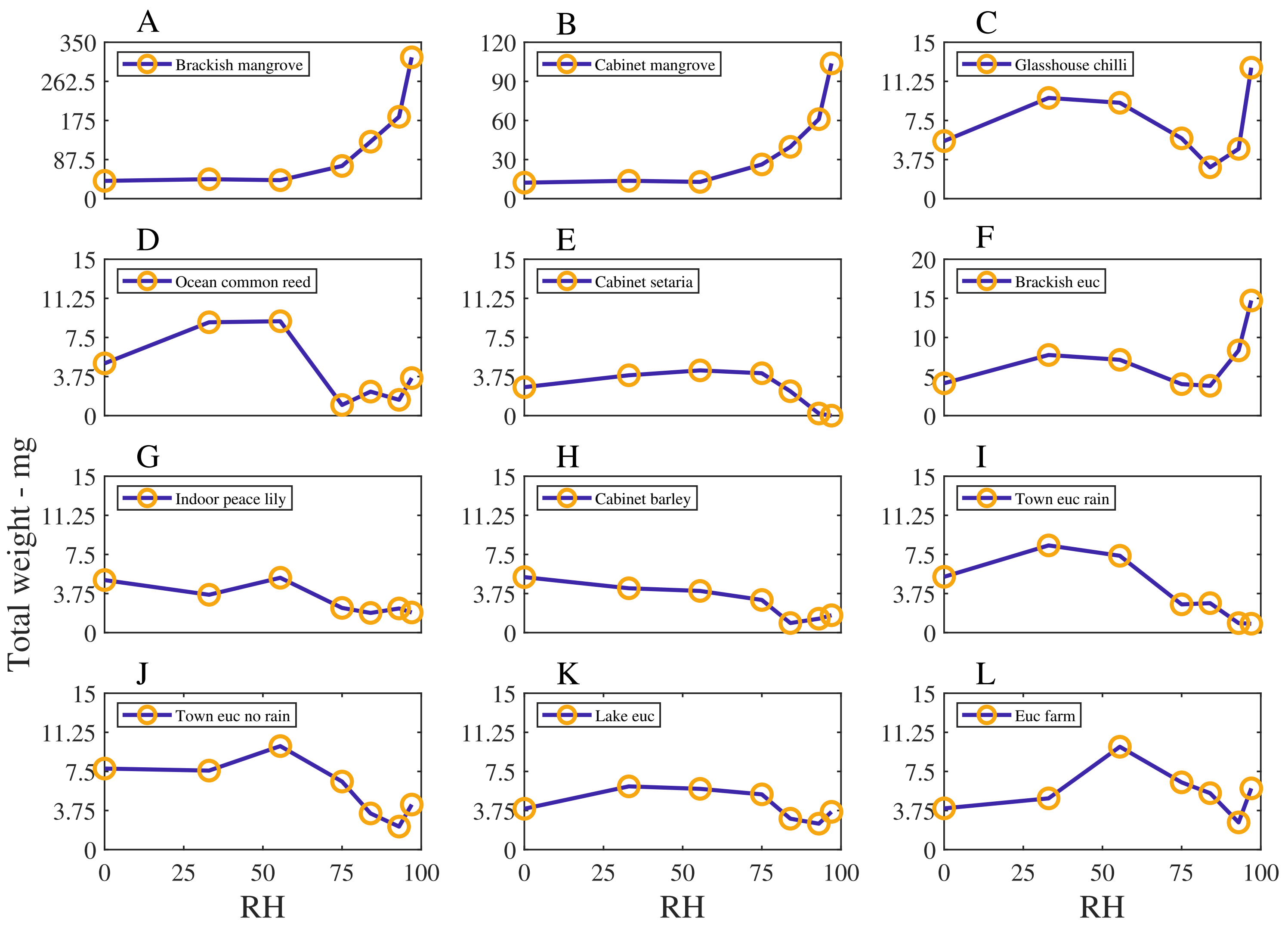

Figure S2: The Total weight (mg) of moisture adsorbed for the leaf wash samples, with relative humidity, RH (%). Total weight includes the dry weight and is scaled with the blank. Note that the *y*-axis range changes for each subfigure.

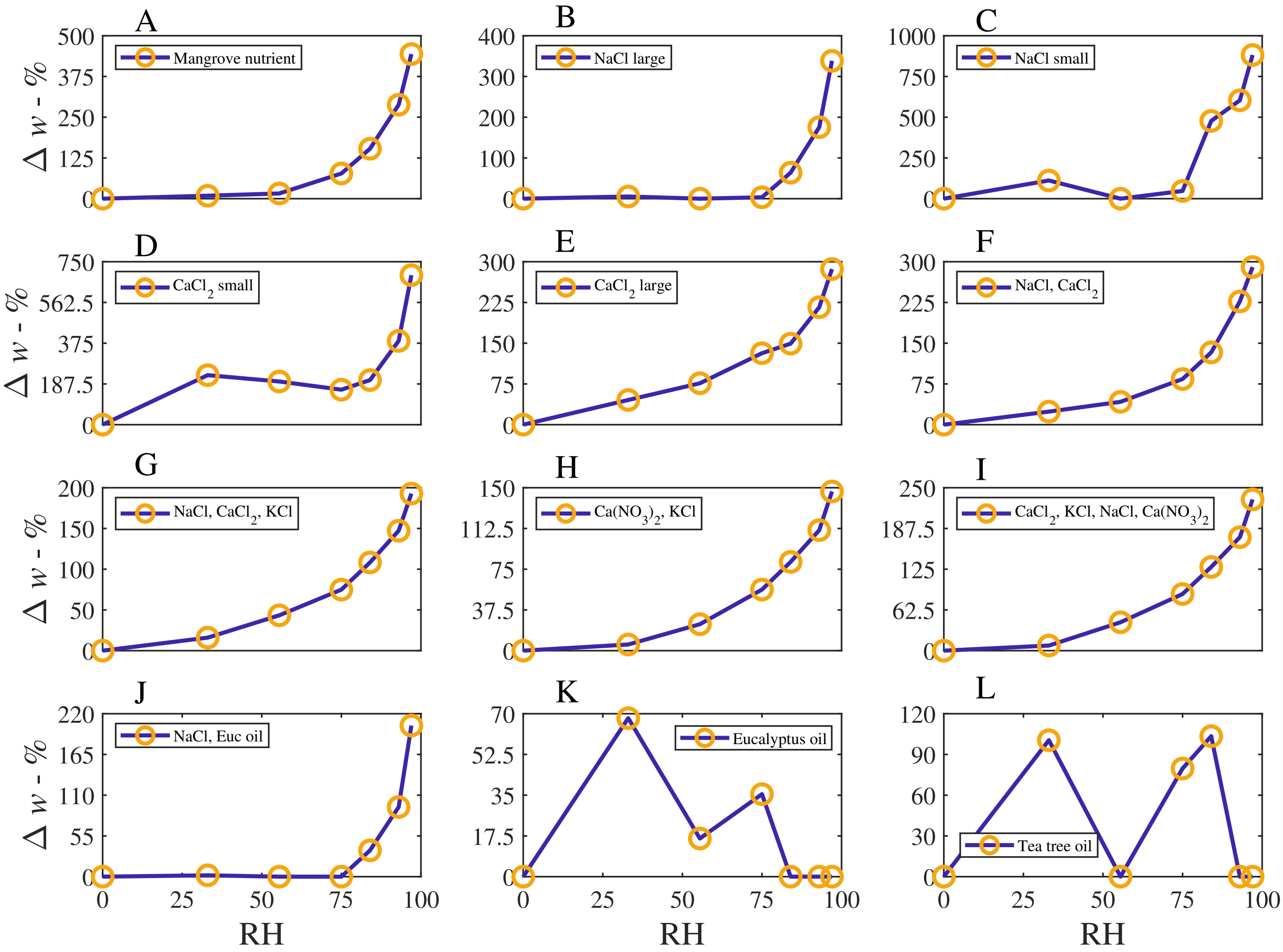

Figure S3: The percentage weight increase of moisture adsorbed above the dry weight of the controls, Δw - %, with relative humidity, RH - %. The y-axis range changes for each subfigure.

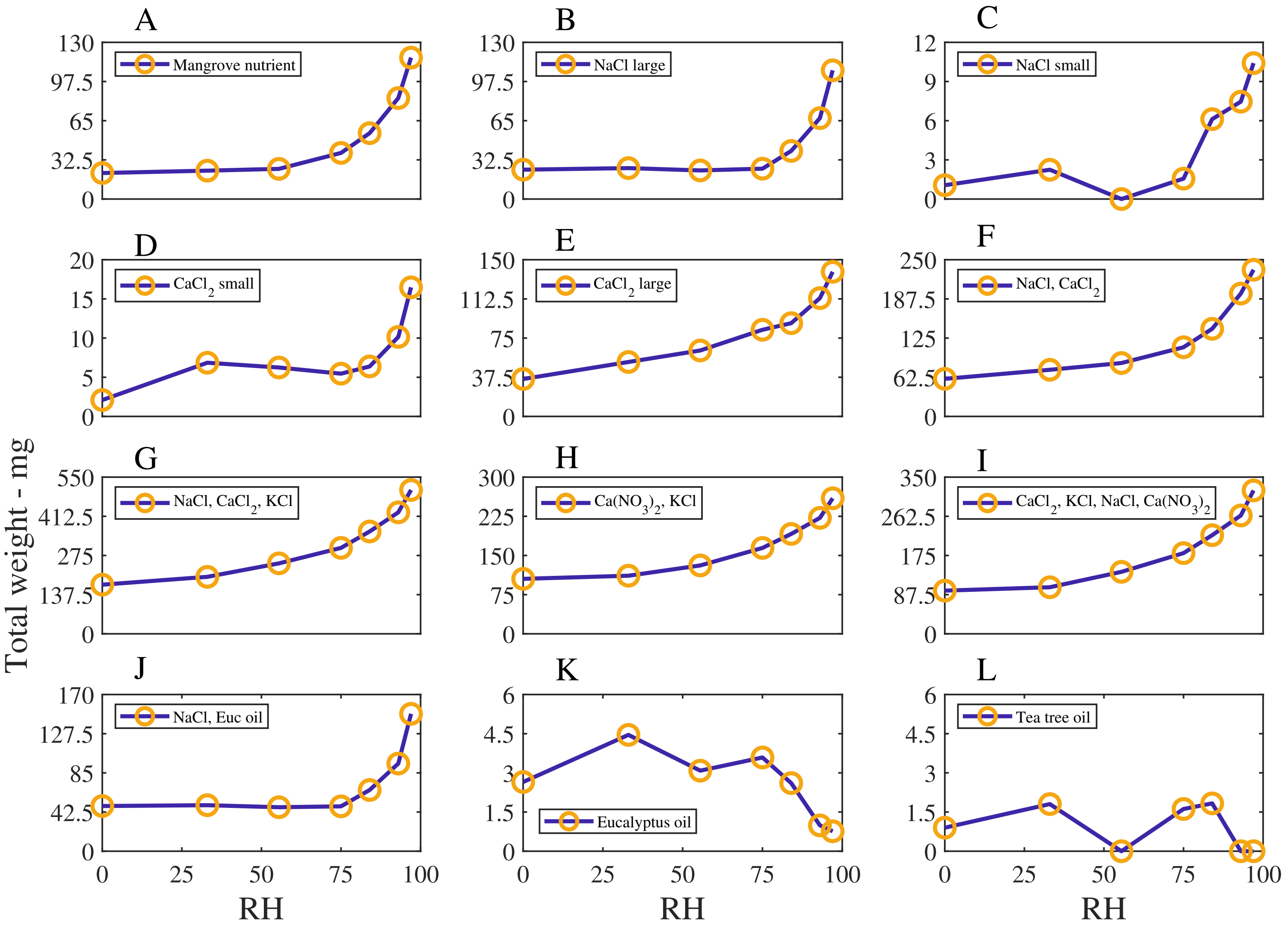

Figure S4: The Total weight (mg) of moisture adsorbed for the controls, with relative humidity, RH (%). The total weight includes the dry weight and is scaled with the blank. The *y*-axis range changes for each subfigure.

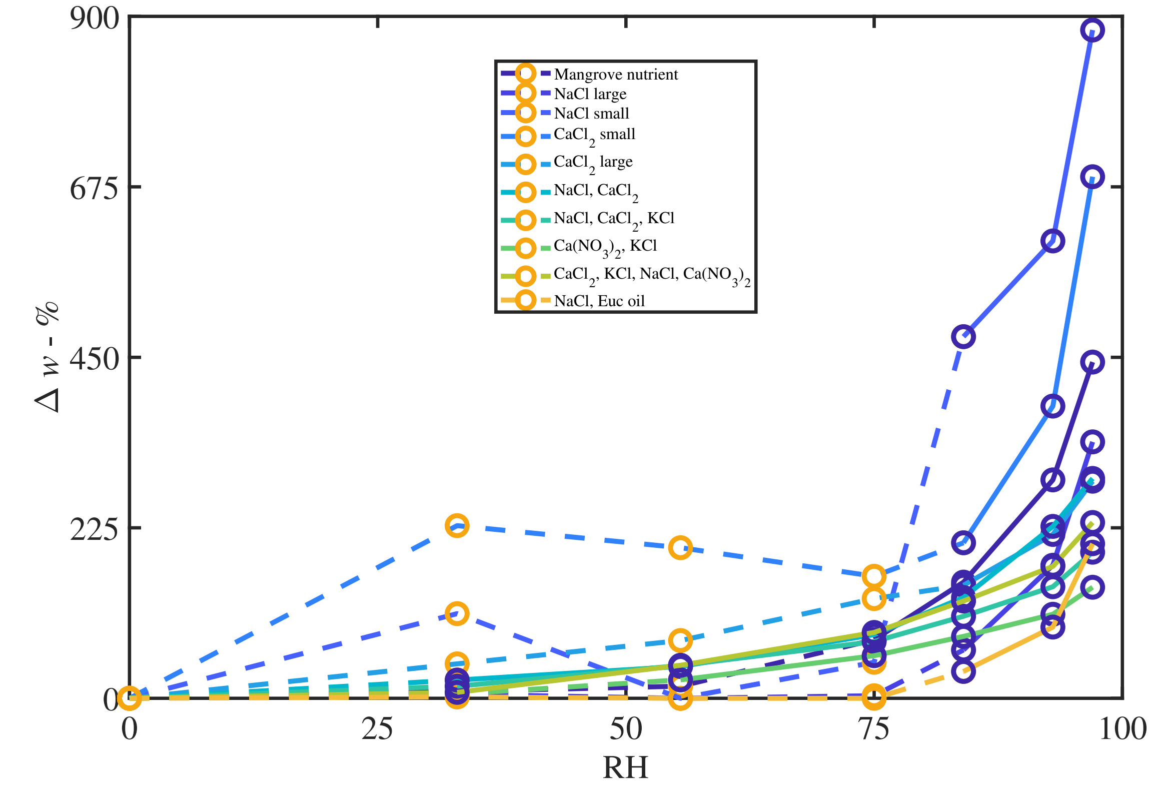

Figure S5: Control samples that have developed a coating of liquid water that is visible without the need for special equipment. The percentage weight increase of moisture adsorbed above the dry weight of the controls, Δw - %, with relative humidity, RH - %, is shown. The orange circles and dashed lines indicate water is not yet visible, and the blue circles and solid lines indicates where liquid water is visible.

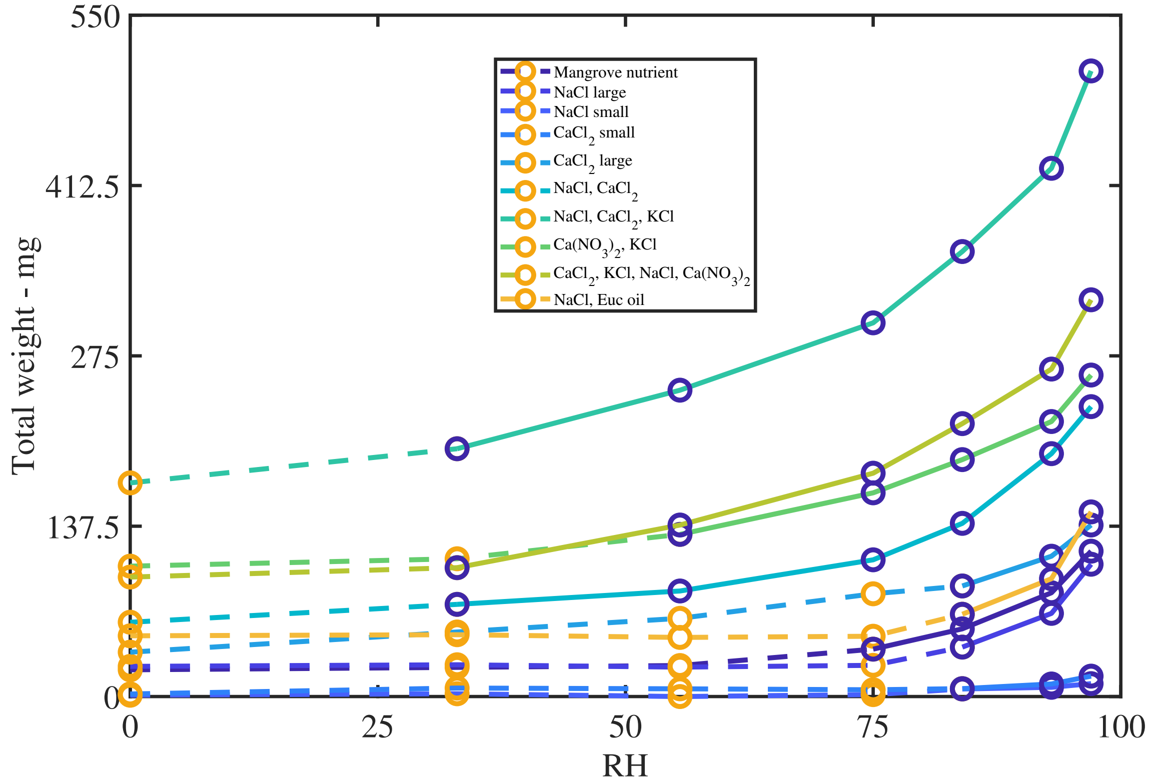

Figure S6: Control samples that have developed a coating of liquid water that is visible without the need for special equipment. The Total weight (mg) of moisture adsorbed for the controls, with relative humidity, RH (%), is shown. The orange circles and dashed lines indicate water is not yet visible, and the blue circles and solid lines indicates where liquid water is visible. Noteworthy is the significant weight of the NaCl + $CaCl_2$ + KCl sample at 97%RH of 500 mg or 0.5 mL, although this sample started with the largest dry weight.

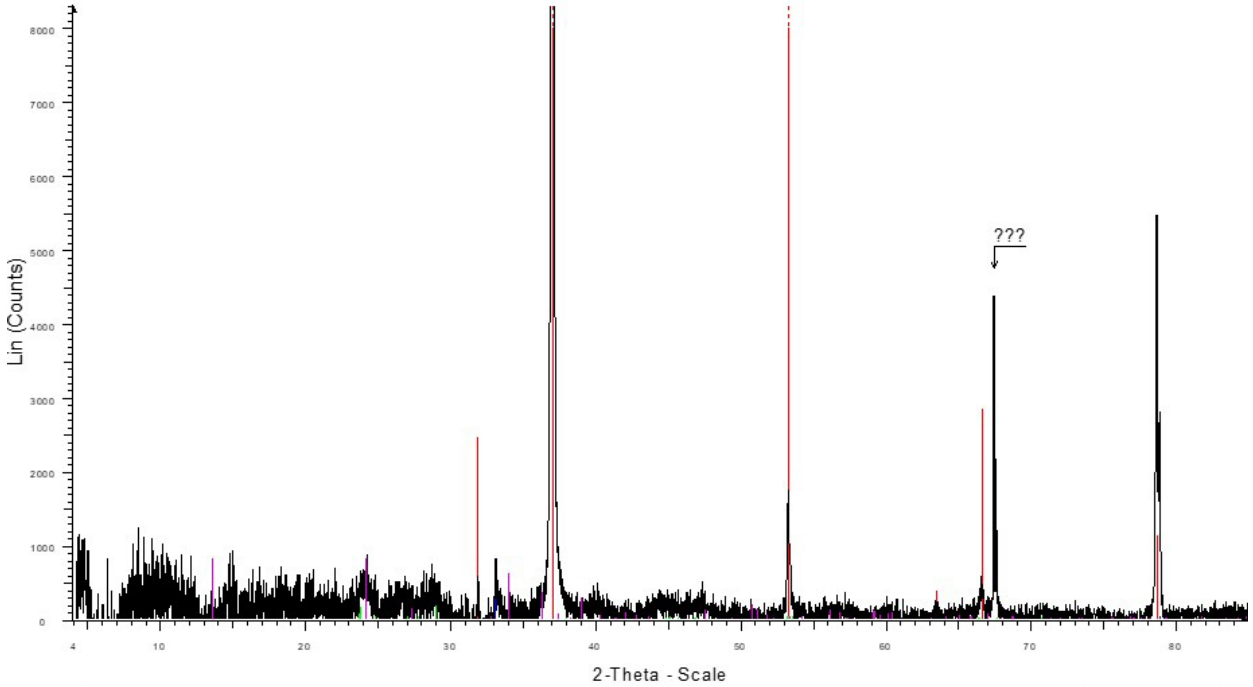

Figure S7: XRD results for Brackish mangrove leaf wash from the liquid wash, and corresponding results are shown in Table 2, with intensity (counts) vs angle (deg 2theta). Red is halite NaCl, blue is sylvite KCl, green is kaolinite $Al_2Si_2O_5(OH)_4$ and magenta is gypsum $CaSO_4$. The question marks refer to an unidentified phase, possibly quartz.

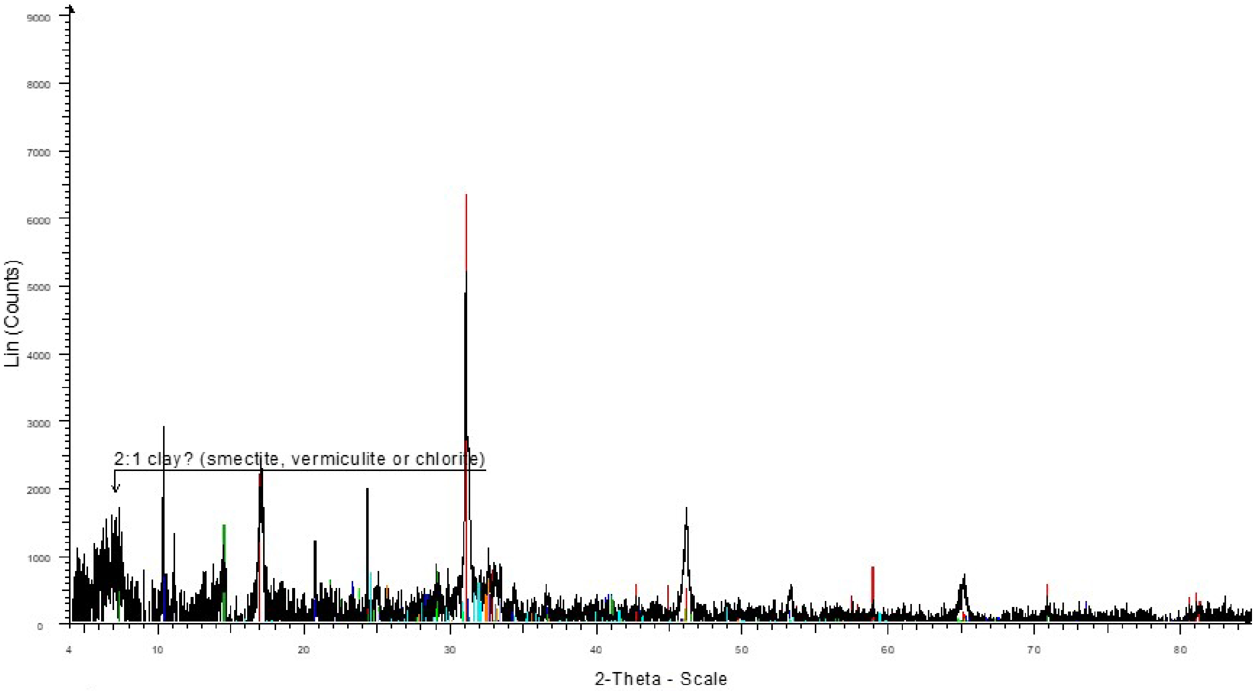

Figure S8: XRD results for Town euc rain leaf wash from the liquid wash, and corresponding results are shown in Table 2, with intensity (counts) vs angle (deg 2theta). Red is quartz SiO_2, blue muscovite, light green is kaolinite $Al_2Si_2O_5(OH)_4$, dark green is chlorite, orange is plagioclase $(Ca,Na)_{1-2}(Si,Al)_{2-3}O_8$, turquoise is K-feldspar $KAlSi_3O_8$, lilac is talc $Mg_3(OH)_2Si_4O_{10}$, grey is sylvite KCl and dark red is boehmite AlOOH.

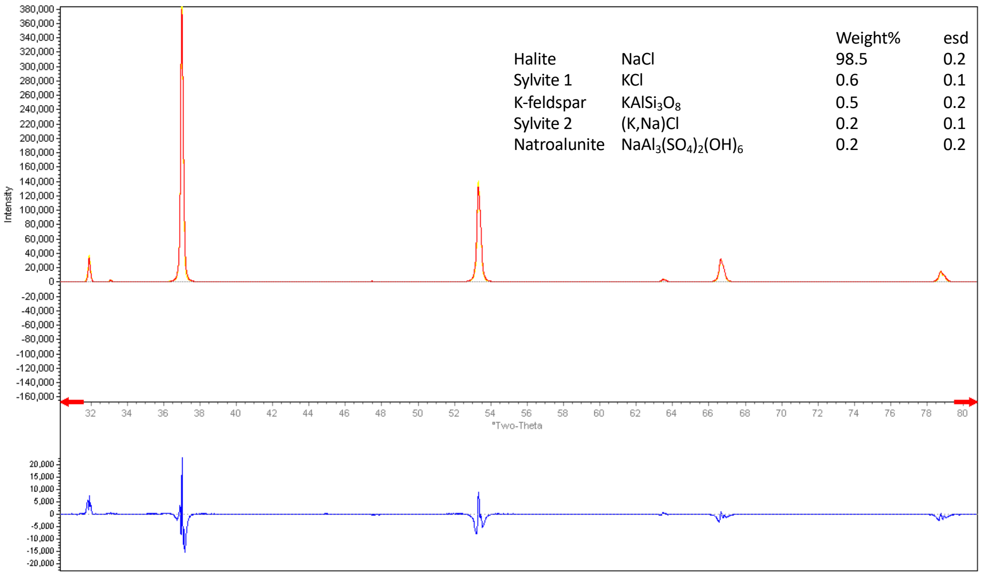

Figure S9: XRD results for a glasshouse mangrove, with intensity (counts) vs angle (deg 2theta). The sample for the XRD was collected by scraping the particles on the bottom of the leaf directly. Note this is the same plant species but a different individual as the Cabinet mangrove sample, and collected at a later date and grown in a glasshouse. No washing of the leaves occurred while the plant was growing.

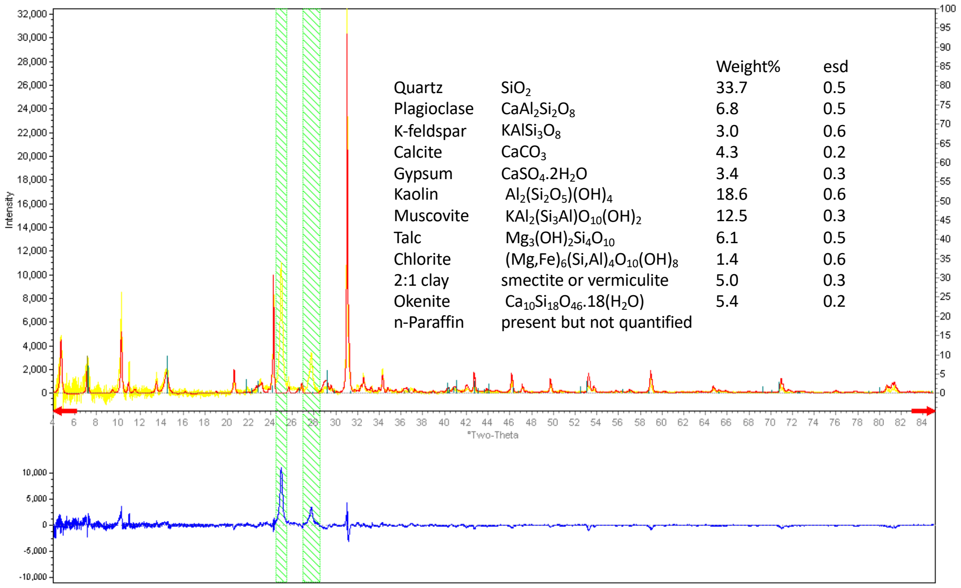

Figure S10: XRD results of Queensland kauri pine (*Agathis robusta* (syn. *A. palmerstonii))*, located indoors, with intensity (counts) vs angle (deg 2theta). This species was not included in the original sample set but was located in the same space as the Indoor peace lily. The plant was mature and had very waxy leaves that had a whitish sheen. Several waxes were seen but not quantified (n-Paraffin). The sample for the XRD was collected by scraping the top of the leaf. The bottom scraping shows similar results to the top, and similar to directly onto the leaf, however scraping showed the best results. Analysis was also conducted on the Town euc no rain and lambs ear (*Stachys byzantina*) with very hairy leaves located next to the town euc, and the results are similar to those shown here.
